# Thrombus Aspirates From Patients With Acute Ischemic Stroke Are Infiltrated by Viridans Streptococci

**DOI:** 10.1161/JAHA.123.030639

**Published:** 2023-11-20

**Authors:** Olli Patrakka, Sari Tuomisto, Juha‐Pekka Pienimäki, Jyrki Ollikainen, Niku Oksala, Vili Lampinen, Markus J. T. Ojanen, Heini Huhtala, Vesa P. Hytönen, Terho Lehtimäki, Mika Martiskainen, Pekka J. Karhunen

**Affiliations:** ^1^ Department of Forensic Medicine, Faculty of Medicine and Health Technology Tampere University and Fimlab Laboratories Tampere Finland; ^2^ Vascular Centre Tampere University Hospital Tampere Finland; ^3^ Department of Neurology Tampere University Hospital Tampere Finland; ^4^ Surgery, Faculty of Medicine and Health Technology Tampere University Tampere Finland; ^5^ Laboratory of Protein Dynamics, Faculty of Medicine and Health Technology Tampere University Tampere Finland; ^6^ Faculty of Social Sciences Tampere University Tampere Finland; ^7^ Department of Clinical Chemistry, Faculty of Medicine and Health Technology Tampere University, Fimlab Laboratories and Finnish Cardiovascular Research Center Tampere Tampere Finland; ^8^ National Institute for Health and Welfare Helsinki Finland

**Keywords:** carotid artery stenosis, oral health, stroke, viridans streptococci, Ischemic Stroke, Inflammation, Pathophysiology, Atherosclerosis

## Abstract

**Background:**

Acute ischemic stroke may be due to embolism from ruptured atherosclerotic carotid arteries. DNA of oral bacteria, mainly the viridans streptococci group, has been detected in thrombus aspirates of patients with ischemic stroke as well as in carotid endarterectomy samples. Because viridans streptococci are known to possess thrombogenic properties, we studied whether their presence in thrombus aspirates and in carotid artery specimens can be confirmed using bacterial immunohistochemistry.

**Methods and Results:**

Thrombus aspirates from 61 patients with ischemic stroke (70.5% men; mean age, 66.8 years) treated with mechanical thrombectomy, as well as carotid endarterectomy samples from 20 symptomatic patients (65.0% men; mean age, 66.2 years) and 48 carotid artery samples from nonstroke autopsy cases (62.5% men; mean age, 66.4 years), were immunostained with an antibody cocktail against 3 species (*Streptococcus sanguinis*, *Streptococcus mitis,* and *Streptococcus gordonii*) of viridans streptococci. Of the thrombus aspirates, 84.8% were immunopositive for viridans streptococci group bacteria, as were 80.0% of the carotid endarterectomy samples, whereas immunopositivity was observed in 31.3% of the carotid artery samples from nonstroke autopsies. Most streptococci were detected inside neutrophil granulocytes, but there were also remnants of bacterial biofilm as well as free bacterial infiltrates in some samples.

**Conclusions:**

Oral streptococci were found in aspirated thrombi of patients with acute ischemic stroke as well as in carotid artery samples. Our results suggest that viridans streptococci group bacteria may play a role in the pathophysiology of ischemic stroke.

**Nonstandard Abbreviations and Acronyms**
ARRIVE: Animal Research: Reporting of In Vivo Experiments
ATCC: American Type Culture Collection
BMG: Brain, Microbes and Genetics
TSDS: Tampere Sudden Death Study
TVS: Tampere Vascular Study

**Research Perspective**

**What Is New?**

- Confirming previous bacterial DNA findings in blood clots of patients with ischemic stroke, oral streptococcal bacteria were found inside neutrophil granulocytes in blood clots and in the wall of symptomatic and in asymptomatic carotid arteries by bacterial immunohistochemistry.

**What Question Should be Addressed Next?**

- Can the outcome of cerebral infarction be affected with a timely short‐term antimicrobial treatment against oral streptococci?
- Could it be possible to develop a safe vaccine against oral streptococci to reduce the risk for an ischemic stroke caused by bacteria‐triggered thrombosis?

Ischemic strokes represent the majority of strokes (85%), and the rest (15%) are hemorrhagic strokes caused by an intracerebral or subarachnoid hemorrhage.^1^ Carotid artery atherosclerosis is an important cause of large vessel stroke, and a portion of acute ischemic strokes may be due to embolism from ruptured carotid arteries.^2^, ^3^, ^4^

Classic risk factors for acute ischemic stroke include age, male sex, hypertension, atrial fibrillation, diabetes, hypercholesterolemia, and smoking, but these do not completely account for the pattern of stroke epidemiology.^5^ Stroke is a common complication in severe bacterial infections, such as endocarditis and meningitis, but it also occurs in other, more common and milder infections.^5^, ^6^ Increased levels of antibodies against several bacteria, such as streptococci, staphylococci, and enterobacteria, were found in 44% of young patients with stroke, but in only 9% of controls.^7^ Peripheral blood neutrophil count is an independent predictor of stroke severity on admission, a greater degree of disability at discharge, and 30‐day mortality.^8^, ^9^ Severe chronic dental infection was found to predispose to stroke in men, possibly by affecting blood coagulation and platelet function.^10^ Moreover, previous infections or chronic exposure to common infective agents are also suggested to contribute to carotid atherosclerosis.^11^

Recent developments in molecular microbiological methods have attracted new interest in studying the involvement of pathogens in cardiovascular diseases. We recently amplified viridans group streptococcal DNA using quantitative polymerase chain reaction from the majority of aspirated thrombi of patients with acute ischemic stroke treated with mechanical thrombectomy (Figure 1).^12^ Subsequently, we reported that these patients with stroke had poor oral health, which tended to associate with carotid stenosis.^13^ Viridans streptococci species belong to the normal oral bacterial microbiome but are also known to cause infective endocarditis and possess thrombogenic properties.^14^, ^15^ DNA of viridans group streptococcal bacteria was also detected in thrombus aspirates from patients with myocardial infarction as well as from patients with lower leg thrombosis due to peripheral atherosclerosis.^16^, ^17^

**Figure 1:**
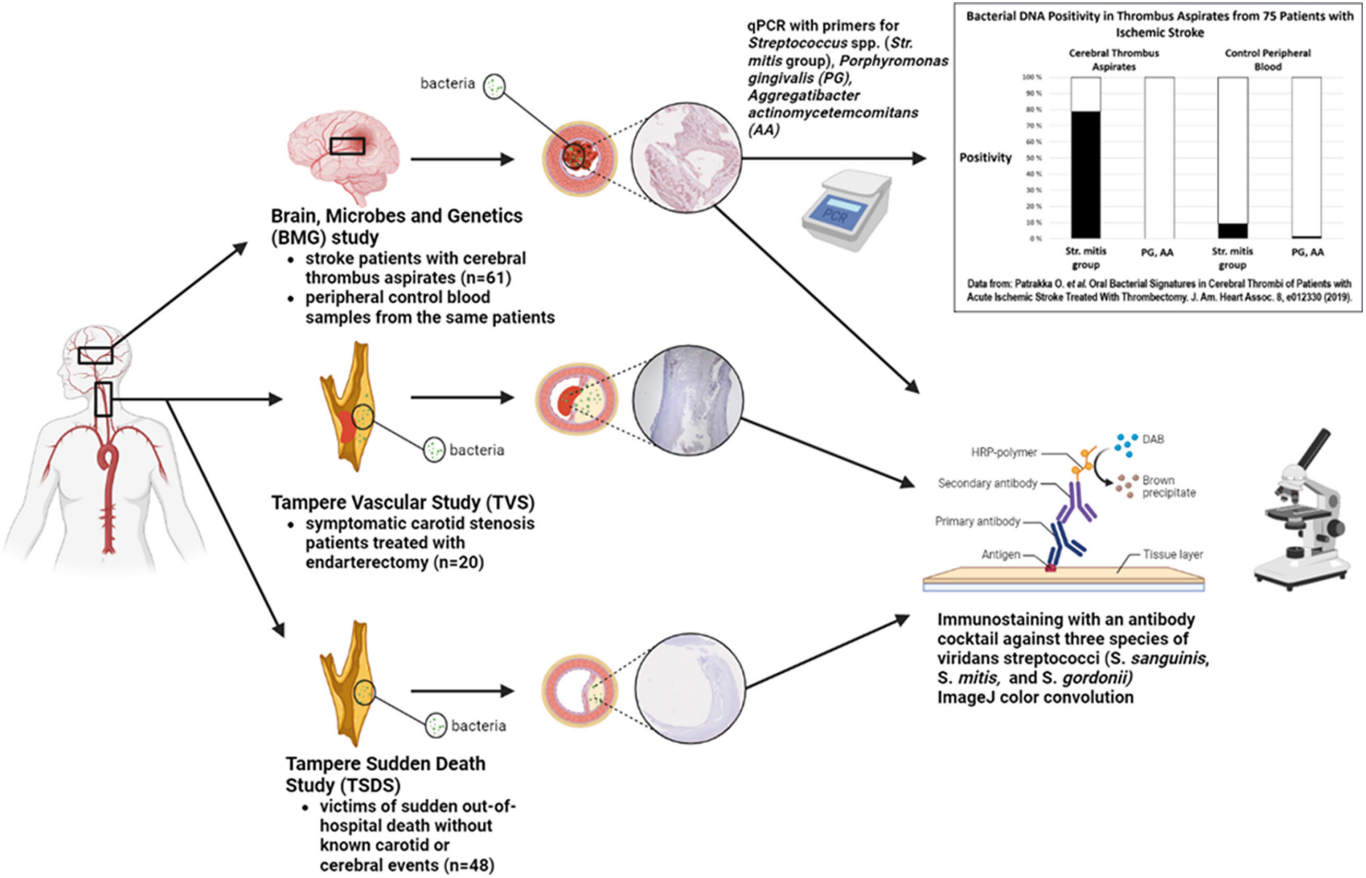
Study protocol. qPCR data modified from Patrakka et al.^12^ Figure created with BioRender.com. DAB indicates 3,3′‐Diaminobenzidine; HRP, horseradish peroxidase; and qPCR, quantitative polymerase chain reaction.

In the current study, we examined whether the presence of viridans streptococci group bacteria can be confirmed in the thrombus aspirates of patients with stroke and in carotid artery specimens using bacterial immunohistochemistry.

## Methods

The data that support the findings of this study are available from the corresponding author upon reasonable request.

The present study is based on a bacterial immunohistochemical study of cerebral thrombus aspirates from patients with acute ischemic stroke in the BMG (Brain, Microbes and Genetics) study, as well as carotid endarterectomy samples from symptomatic patients in TVS (Tampere Vascular Study) and carotid artery samples from nonstroke autopsy cases in TSDS (Tampere Sudden Death Study) (Figure 1).

### BMG Study

Thrombus aspirates were collected from 61 consecutive patients with ischemic stroke who had large‐vessel occlusion, mainly middle cerebral artery M1 and M2 segment, (mean age, 66.8 years; 70.5% men), treated with mechanical thrombectomy between December 2013 and July 2016 in the Acute Stroke Unit of Tampere University Hospital, Tampere, Finland. The thrombus was aspirated from the middle cerebral artery in 60 of the 61 samples (98.4%) of the cases, with 1 thrombus aspirated from the vertebrobasilar artery. Patients with thrombolysis in cerebral infarction 0 or 1 recanalization grade were excluded as the thrombus was unsuccessfully retrieved. A neurologist examined all patients when they arrived at the hospital and evaluated the possibility of revascularization using thrombectomy together with a neurointerventional radiologist. According to the TOAST classification, the cause of large‐vessel occlusions of the brain in patients treated with endovascular thrombectomy during the study period in Tampere University Hospital was cardioembolic in 38% and atherosclerotic in 62% of the cases (personal communication, Dr Jyrki Ollikainen).^18^ The median delay between the onset of an ischemic stroke and hospital arrival was 2 hours and 20 minutes (range, 0–16 hours). Medical history was collected from Tampere University Hospital digital patient archives. None of the patients had been treated with antibiotics or had experienced severe infections or septicemia during the stroke.

During mechanical thrombectomy, an introducer sheath was placed into a femoral artery, and a control blood sample for bacterial genetic analysis was collected through the sheath. A guiding catheter of up to 9F (Merci, Stryker Neurovascular) with a tip balloon was then navigated into the carotid artery proximally to the occluded site. The microcatheter (0.021") with the guidewire was used to navigate through the occluded site and to deploy the stent retriever (Trevo, Stryker Neurovascular) over the thrombus. An additional distal access catheter was used to reach the thrombus if needed. Forceful aspiration through proximal catheters was achieved with a 60‐mL syringe while retrieving the deployed stent. In a minority of the cases, only direct thrombus aspiration was used. The device settings were selected by the operator case by case. Thrombectomy was repeated until the angiologic result was satisfactory. The gathered thrombus was divided into a segment placed in a 1.5‐mL microcentrifuge tube for quantitative polymerase chain reaction analysis and a histological sample placed in a formalin container.

### Tampere Vascular Study

Endarterectomy samples were collected from 20 symptomatic patients (mean age, 66.2 years; 65% men) diagnosed with ipsilateral carotid artery stenosis. All open vascular surgical procedures were performed at the Division of Vascular Surgery and the Heart Center at Tampere University Hospital between 2006 and 2009. The patients had experienced amaurosis fugax, a transient ischemic attack, or a stroke. Eight (40%) patients had an acute ischemic stroke. The severity of the carotid stenosis was histologically classified, the average being 82% (range, 69%–99%). All samples were classified as either fibrotic or calcified atheroma (American Heart Association type V) or complicated atheroma with rupture and thrombosis/hemorrhage (American Heart Association type VI) according to the American Heart Association classification.^19^, ^20^ Medical history was collected from Tampere University Hospital digital patient archives.

In the study, a small, 2×2‐ to 3‐mm endarterectomy sample that could be extracted without endangering the patient was immediately placed in sterile 10% buffered formalin. From the initial 96 samples obtained for histology, 20 (20.8%) were selected because they were large enough for immunohistochemical studies.

### Tampere Sudden Death Study

Postmortem carotid artery samples were collected from 48 autopsy cases (mean age, 66.4 years; 62.5% men) during medicolegal autopsies at the Department of Forensic Medicine at the University of Tampere between 2010 and 2015, representing a cross‐section of the population. The selection criteria for the cases were out‐of‐hospital death, time elapsed postmortem of <6 days, intact middle torso and bowel, no signs of bacterial infections or drug addiction, and no visible wounds or necrosis. None of the patients had died of stroke.

The time interval between death and storage of the body in the mortuary was <24 hours in all cases. In the mortuary, the bodies were kept at 4 °C. Low temperatures prevent bacterial growth, and bacterial populations are unlikely to alter during such storage conditions.^21^ Based on hospital records, incident police reports with data on drugs found in the home and possible treatments, as well as physician's admission notes, none of the patients with a sudden out‐of‐hospital death had used antibiotics within 2 weeks prior to their death. The relatives of the deceased were sent a structured interview concerning risk factors and other background information.

### Ethics

All participating patients gave their written consent to the study. TVS was approved by the ethics committee of Pirkanmaa Hospital District (R99204). All clinical investigations were conducted according to the principles of the Declaration of Helsinki. The BMG study was approved by the ethics committee of Pirkanmaa Hospital District (R13093), and the study was explained to the patients. In TSDS, informed consent was given by relatives. TSDS was approved by the ethics committee of Pirkanmaa Hospital District (R09097). Methods were reported according Animal Research: Reporting of In Vivo Experiments (ARRIVE) guidelines.^22^

### Quantitative Polymerase Chain Reaction

Bacterial DNA was extracted from the BMG samples using a commercially available QIAamp DNA Mini Kit (Qiagen) according to the instructions provided. Quantitative polymerase chain reaction was used to detect the presence of bacterial DNA in the thrombus and blood samples from the same patients by using published oligonucleotide primers and probes for *Streptococcus* spp., mainly the *Streptococcus mitis* group, as well as *Porphyromonas gingivalis* and *Aggregatibacter actinomycetemcomitans*. Universal bacterial primers and probe were determined with RNaseP (Applied Biosystems) as a reference. These methods are described in detail elsewhere.^12^

### Immunohistochemistry

Antibodies against 3 major viridans streptococcal species—*Streptococcus sanguinis* (American Type Culture Collection [ATCC] 10556), *S mitis* (ATCC 49456), and *Streptococcus gordonii* (ATCC 10558)^12^—were produced by ThermoFisher using 90‐day rabbit immunization protocol. The performance of the resulting antibodies was confirmed with Western blot by staining the respective streptococcal lysates (data not shown). All histological samples were immunostained with an antibody cocktail containing antibodies raised against the 3 species of viridans streptococci. The immunostaining intensity was assessed with a semiquantitative score (+/++/+++). ImageJ software (github.com/fiji/fiji) was used to create color convolution in order to increase the contrast in the immunostained samples for better visualization of the results. Control stainings were performed with an IgG isotype control antibody (ab37415, Abcam) as well as with an IgG anti–*Escherichia coli* antibody (ab137967, Abcam) raised in rabbits. The specificities of the antibodies were tested in a histological tissue section by inoculating them with a suspension of streptococcal bacteria species (obtained from ATCC) and performing an immunohistochemical study using an antibody against the same bacteria (Figure S1). Gut and liver samples from the TSDS autopsy cases were stained with a streptococcal cocktail to show the ability of the antibodies to detect streptococci in feces as well as in liver Kupffer cells (Figure S2).

### Statistical Analysis

Statistical analysis was performed using IBM SPSS Statistics 28. Associations between bacterial immunohistochemistry and sample frequencies were analyzed using χ^2^ test. *P*<0.05 was considered statistically significant.

## Results

The mean age and sex of the participants did not differ between the 3 study groups (Table). Cerebrovascular disease was less frequent, and coronary heart disease was more common in the TSDS series. Patients with symptomatic carotid stenosis in the TVS series were more often smokers compared with the other groups. Hypertension was reported more frequently as a risk factor in symptomatic BMG and TVS series patients compared with the TSDS autopsy series.

**Table .** Clinical Characteristics of the Study Series

Characteristics	BMG study (N=61)	TVS (N=20)	TSDS (N=48)
Age, mean±SD, y	66.8±10.9	66.2±8.42	66.4±15.6
Men, n (%)	43 (70.5)	13 (65.0)	30 (62.5)
Diabetes, n (%)	11 (18.0)	4 (20.0)	7 (14.6)
Dyslipidemia, n (%)	24 (39.3)	17 (85.0)	N/A
Arterial hypertension, n (%)	31 (50.8)	15 (75.0)	15 (31.3)
Coronary heart disease, n (%)	13 (21.3)	6 (30.0)	26 (54.2)*
Cerebrovascular disease, n (%)	61 (100)	19 (95.0)	2 (4.2)*
Atrial fibrillation, n (%)	39 (63.9)	N/A	N/A
Heart failure, n (%)	9 (14.8)	1 (5.00)	N/A
Smoking status, n (%)	13 (36.1)†	15 (75.0)	18 (37.5)

BMG indicates Brain, Microbes and Genetics; N/A, data not available; TSDS, Tampere Sudden Death Study; and TVS, Tampere Vascular Study.

* Cause of death.

^†^ Data available from 36 patients.

As regards the aspirated cerebral artery thrombi of patients with stroke, DNA from *Streptococcus* spp., mainly the *S mitis* group, was found in 78.7% of the cases participating in the BMG study (Figure 1), whereas DNA from *P gingivalis* and *A actinomycetemcomitans* was not detected. Of the arterial blood control samples collected during the thrombectomy procedure, 9.33% were positive for *Streptococcus* spp. and 1.33% for both *P gingivalis* and *A actinomycetemcomitans*. These findings have been described in detail in a previous article.^12^

**Figure 2:**
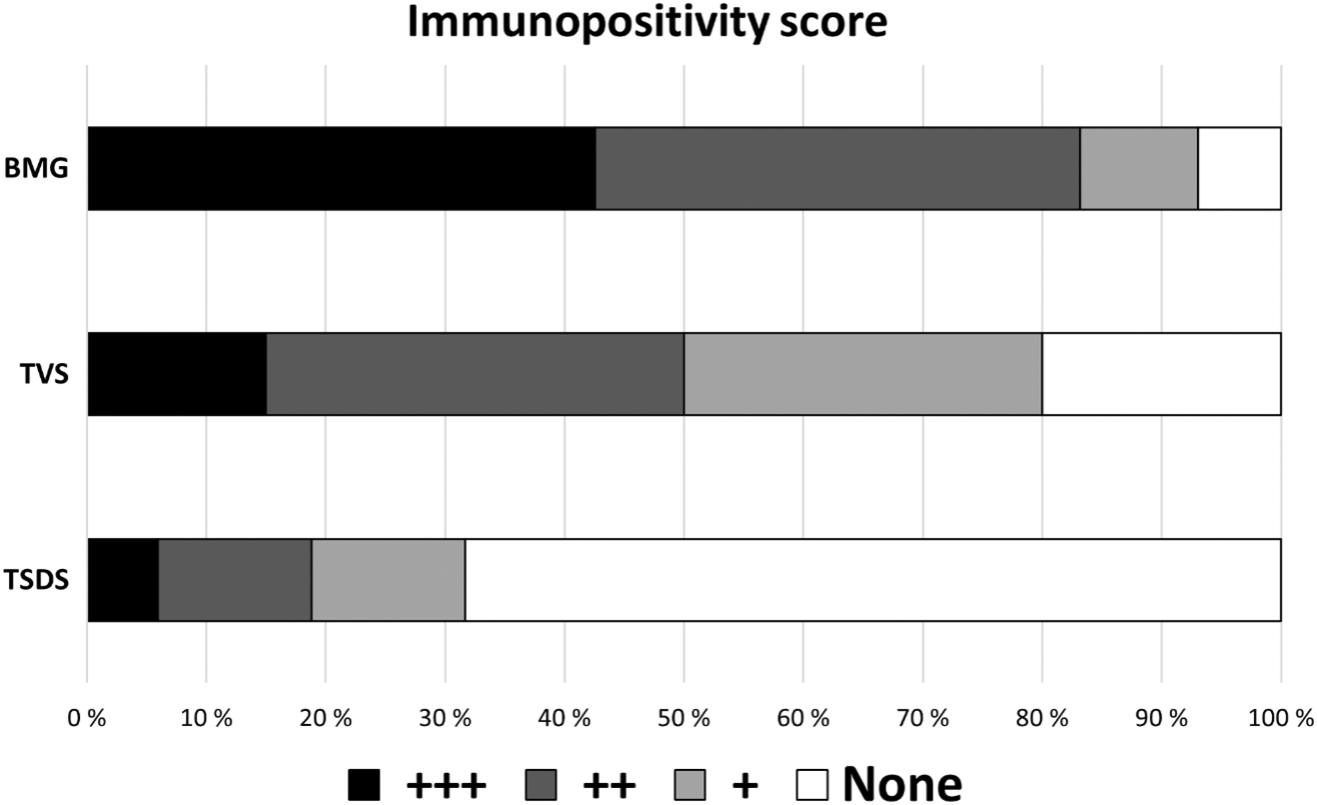
Sample frequencies and a 3‐step classification of intensity of immunopositivity (*P*<0.001). BMG indicates Brain, Microbes and Genetics; TSDS, Tampere Sudden Death Study; and TVS, Tampere Vascular Study.

**Figure 3:**
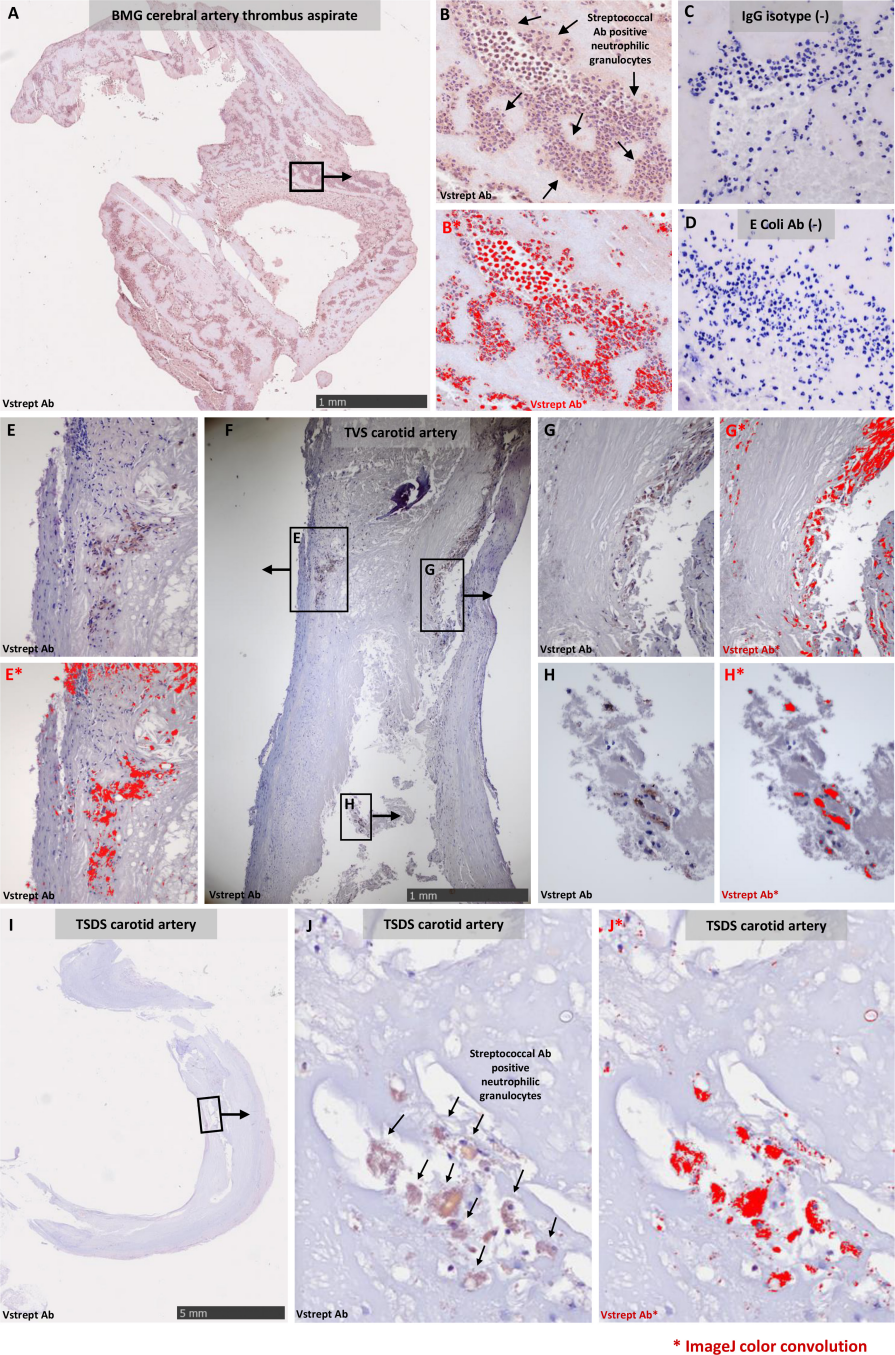
Bacterial immunostaining of the samples. Viridans streptococcal immunostaining showing diaminobenzidine (brown) and color convoluted (red) microscopical photographs of bacterial infiltrates in thrombus aspirates from the BMG series (**A** and **B**), ruptured carotid atheroma samples from TVS (**E** through **H**), and carotid artery samples from the TSDS series (**I–J**). Thrombus aspirates were also stained with IgG isotype (**C**) and *Escherichia coli* (**D**) antibodies to show the specificity of the bacterial immunostaining. BMG indicates Brain, Microbes and Genetics; N/A, data not available; TSDS, Tampere Sudden Death Study; and TVS, Tampere Vascular Study.

Of the 61 thrombus aspirate samples from the patients with stroke in the BMG study, 39 (84.8%) were histologically positive (+/++/+++) for viridans streptococci group bacteria (Figure 2). Most streptococci were detected inside neutrophil granulocytes, but there were also remnants of bacterial biofilm as well as free bacterial infiltrates in some samples (Figure 3). High numbers of neutrophilic granulocytes were present in the samples.

Most (80.0%) carotid endarterectomy samples obtained from symptomatic patients included in TVS were positive for oral streptococci (Figure 2). In contrast, the carotid artery samples from the TSDS study comprising out‐of‐hospital deaths, mainly due to coronary heart disease, showed considerably less (31.3%) immunopositivity, with 16.7% of the samples being clearly immunopositive (++/+++). In the TVS endarterectomies and TSDS carotid artery sample series, oral streptococci were found in clusters of streptococcal antibody‐positive neutrophilic granulocytes as well as remnants of bacterial biofilm (Figure 3). The immunopositivity scores were highest in the BMG thrombus aspirates and TVS carotid endarterectomies (*P*<0.001) (Figure 2).

Staining of the samples with an IgG isotype antibody or *E coli* antibody resulted in a negligible signal, suggesting the reliability of the viridans streptococcal antibody cocktail staining (Figure 3).

## Discussion

The decline in strokes during the 20th century can only incompletely be explained by conventional risk factors and their temporal trends.^5^, ^23^ It has been long suspected that bacterial infection may play a role in the pathogenesis of stroke.^6^ Using a molecular microbiological approach, we have found that thrombus aspirates from patients with stroke contained viridans streptococcal DNA fragments.^12^ In the present study, we confirmed the presence of viridans streptococcal bacteria using immunohistochemistry. Almost all of the thrombus aspirates contained oral streptococci, located mainly inside neutrophilic granulocytes. In symptomatic patients subjected to carotid endarterectomy, most carotid samples were strongly immunopositive for viridans group streptococci. We also found clear streptococcal immunopositivity in 17% of carotid artery samples from autopsy cases representing a cross‐section of the population. To our knowledge, only 1 study has previously reported the presence of viridans group streptococci in carotid artery endarterectomies using immunolocalization,^24^ but the presence of viridans group streptococci in cerebral thrombi has not been previously shown. Our results suggest that there may be a connection between symptomatic inflammation in carotid arteries and cerebral thrombus.

The present concept is that atherosclerosis is a complex chronic inflammatory disorder in the arterial intima, driven by oxidized or otherwise modified low‐density lipoprotein.^25^, ^26^, ^27^ Increasing knowledge suggests that the atherosclerotic process may also be accelerated by a bacterial infection, with infections of oral origin being extensively studied.^28^, ^29^, ^30^, ^31^, ^32^ Both oxidized low‐density lipoprotein and bacteria activate Toll‐like receptor–mediated pathways, leading to the elevation of cytokine levels and systemic inflammation, which are related to the initiation, development, and rupture of an atherosclerotic plaque.^33^, ^34^, ^35^, ^36^ The immune responses that have evolved to combat bacterial infections are thus shared with those involved in the immune response to the inflammatory components of atherogenesis.^37^ Oral infections have also been suggested to contribute to carotid artery intima‐media thickness, leading to carotid artery stenosis and subclinical atherosclerosis,^38^ and, after adjusting for conventional risk factors, a significant association has been reported between the level of tooth loss (as a marker of past periodontal disease) and the prevalence of carotid artery plaques.^39^

Oral bacteria can enter the bloodstream after dental procedures (eg, root canal treatment or tooth extraction) causing transient bacteremia. In tooth extraction, most bacteria translocated into the circulation have been found to be viridans streptococci.^40^, ^41^ Koren et al found that the abundance of viridans streptococcal DNA in atherosclerotic plaques correlated with their abundance in the oral cavity.^42^ After accessing systemic circulation, bacteria might end up in an atherosclerotic lesion via a circulating macrophage or directly through neovasculature developing inside the carotid atherosclerotic plaque.^43^ Viridans streptococci can directly interact with platelets, causing their activation.^15^ Activated platelets (ie, platelet‐derived growth factor) induce the recruitment of proatherosclerotic cells, which speeds up the atherosclerotic processes.^44^, ^45^, ^46^ Following bacteremia, bacteria‐induced thrombi might thus also be trapped in the left atrial appendage, causing a cardioembolic stroke in patients with altered hemodynamics, such as in atrial fibrillation.^45^, ^47^ In our BMG series, 63.9% of the patients had atrial fibrillation. However, despite that, 62% of the cases were considered to be due to atherosclerosis, suggesting uncertainty in determining the cause.

Oral bacteria are known to form biofilms on hard tooth surfaces but also on soft epithelial tissue.^48^ Viridans streptococci are the initial colonizers in the development of a dental plaque.^49^ It has been suggested that bacteria form biofilm‐like structures in the atherosclerotic plaque.^50^, ^51^ This could contribute to the persistent inflammation associated with atherosclerosis. Biofilm‐phenotype bacteria are much more resistant to antibiotics than the same bacteria in planktonic form.

To validate the specificity of the immunohistochemical stainings, we used several controls. The rabbit IgG isotype control is a primary antibody that lacks specificity to the target. Isotype controls are used as negative controls to differentiate a nonspecific background signal from a specific antibody signal. An *E coli*–specific antibody was used to show the specificity of the viridans streptococci antibody cocktail. All IgG isotype control–stained samples as well as the *E coli* immunostainings were negative.

The limitations of our study include the fact that we used 3 different sets of patient samples and that the endarterectomy samples and thrombus aspirate samples were taken from different patients. This cross‐sectional study is a hypothesis‐generating study and cannot demonstrate any etiological relation. In the future, it would be interesting to collect the samples from the same patients. We only used an antibody cocktail against viridans streptococci species, and it is possible that other bacteria may also be involved in the pathogenesis of thrombosis. To demonstrate the cause, a prospective multicenter study with larger patient series should be designed.

## Conclusions

We verified using immunohistochemistry that streptococci can be found in the aspirated thrombi of patients with acute ischemic stroke as well as in atherothrombotic symptomatic carotid artery plaque samples, suggesting their possible involvement in the pathogenesis of ischemic stroke.

## Sources of Funding

This study was supported by grants from the Ida Montin Foundation (O.P.); The Finnish Medical Foundation (O.P.); the Competitive Research Funding of Tampere University Hospital (grant X51001 for T.L. and P.K.); the Emil Aaltonen Foundation (T.L.); the Academy of Finland (grants 286284 and 322098 for T.L. as well as 331946 for V.P.H.); the Tampere Tuberculosis Foundation (T.P. and T.L.); the Finnish Foundation for Cardiovascular Research (grants for T.L., MJ.T.O., and P.K.); the Sigrid Juselius Foundation (grant for V.P.H.); the Yrjö Jahnsson Foundation, Pirkanmaa Regional Fund of the Finnish Cultural Foundation (S.T.); the Elli and Elvi Oksanen Foundation, Pirkanmaa Regional Fund of the Finnish Cultural Foundation (M.J.T.O and V.L.); the European Union 7th Framework Programme (grant 201668 for AtheroRemo); and the European Union Horizon 2020 (grant 755320 for TAXINOMISIS and grant 848146 for To Aition).

## Disclosures

None.

## Supporting information

Figures S1–S2
References^52^, ^53^, ^54^
