# Supplementary material for: Thrombus Aspirates From Patients With Acute Ischemic Stroke Are Infiltrated by Viridans Streptococci
Source: J Am Heart Assoc. 2023 Nov 20;12(22):e030639. doi: 10.1161/JAHA.123.030639 (PMC10727284; doi:10.1161/JAHA.123.030639)
Supplement: Supplementary file 1 — Figures S1–S2 References 52 , 53 , 54 [file JAH3-12-e030639-s001.pdf]

SUPPLEMENTAL MATERIAL

Figure S1. Antibody stainings of the corresponding streptococcal bacteria.

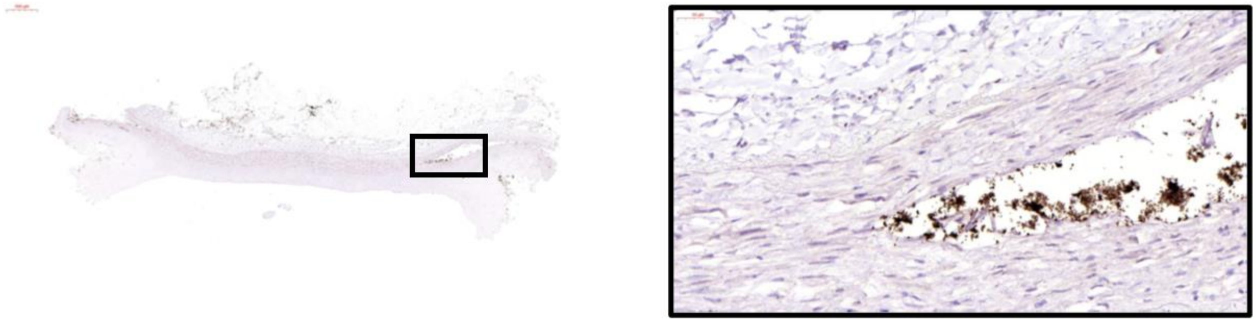

Streptococcus gordonii AB 1:500

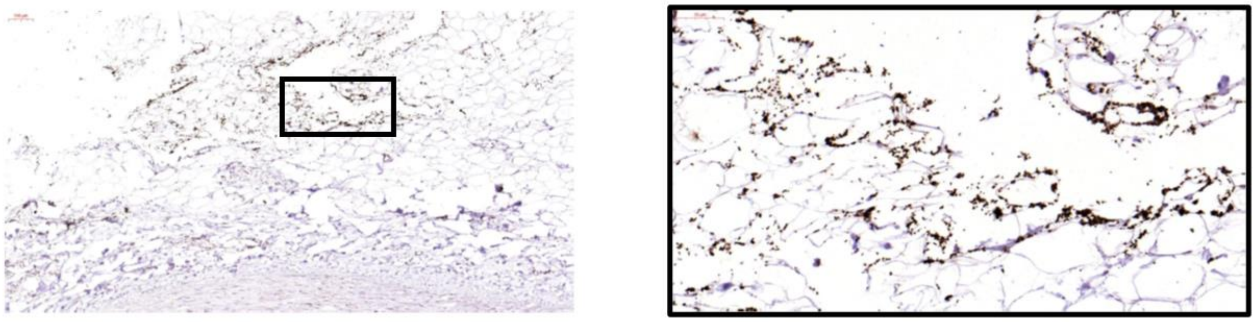

Streptococcus mitis AB 1:500

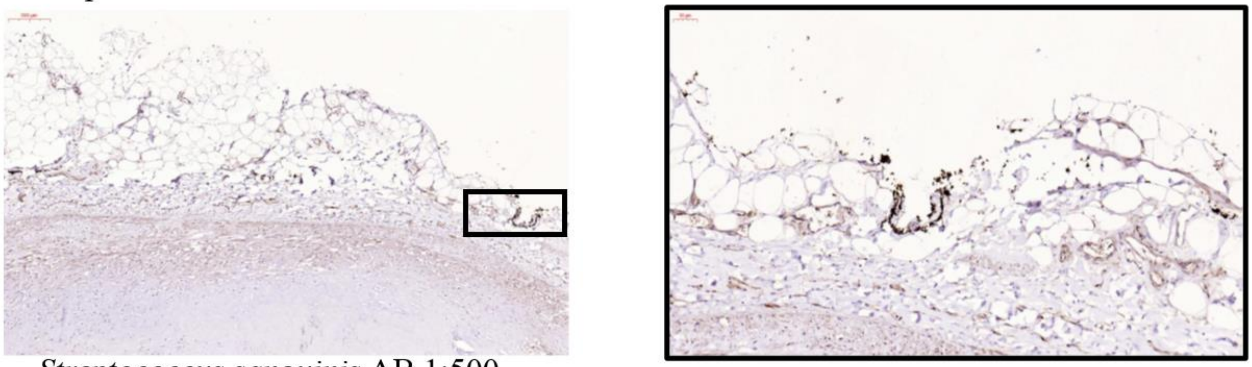

Streptococcus sanguinis AB 1:500

The specificities of the antibodies raised in rabbits (ThermoFischer®) were tested in formalin-fixed histological vascular tissue sections by inoculating (+37 C, 5 hours) them with suspension of ATCC streptococcal bacteria species and performing an immunohistochemical study using antibody against the same bacteria. All antibodies stained corresponding ATCC bacteria intensively with no (*S. gordonii* and *S. mitis*) or weak (*S. sanguinis*) background.

AB; antibody.

Figure S2. Viridans streptococcal (Vstrept Ab) immunostaining of gut and liver samples.

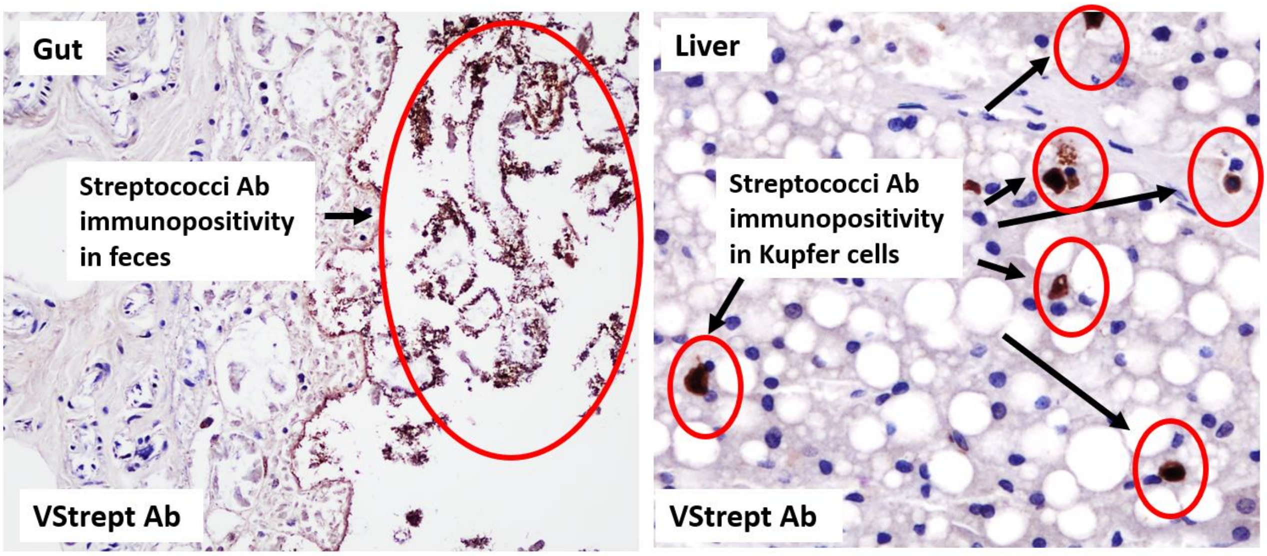

To test whether viridans streptococcal antibody cocktail also stains streptococci present in gut content and in liver Kupfer cells, we immunostained samples from Tampere Sudden Death Study using the same concentration as was used for thrombus aspirates and carotid artery sample stainings.

Viridans streptococcal immunostaining revealed masses of streptococci in gut as well as strong and specific positivity in liver Kupfer cells.

Viridans group streptococci are part of a normal gut microbiome colonizing the gastrointestinal and genitourinary tracts as well as the oral mucosa[52]. The liver is the first line of defense against a continuously occurring influx of microbially derived products and bacteria from the gut[53,54].

Ab; antibody.
